# An MRI-based pelvimetry nomogram for predicting surgical difficulty of transabdominal resection in patients with middle and low rectal cancer

**DOI:** 10.3389/fonc.2022.882300

**Published:** 2022-07-25

**Authors:** Yuan Yuan, Dafeng Tong, Minglu Liu, Haidi Lu, Fu Shen, Xiaohui Shi

**Affiliations:** ^1^ Department of Radiology, Changhai Hospital, Shanghai, China; ^2^ Department of Colorectal Surgery, Changhai Hospital, Shanghai, China

**Keywords:** MRI, surgery, pelvimetry, nomogram, rectal cancer

## Abstract

**Objective:**

The current work aimed to develop a nomogram comprised of MRI-based pelvimetry and clinical factors for predicting the difficulty of rectal surgery for middle and low rectal cancer (RC).

**Methods:**

Consecutive mid to low RC cases who underwent transabdominal resection between June 2020 and August 2021 were retrospectively enrolled. Univariable and multivariable logistic regression analyses were carried out for identifying factors (clinical factors and MRI-based pelvimetry parameters) independently associated with the difficulty level of rectal surgery. A nomogram model was established with the selected parameters for predicting the probability of high surgical difficulty. The predictive ability of the nomogram model was assessed by the receiver operating characteristic (ROC) curve and decision curve analysis (DCA).

**Results:**

A total of 122 cases were included. BMI (OR = 1.269, *p* = 0.006), pelvic inlet (OR = 1.057, *p* = 0.024) and intertuberous distance (OR = 0.938, *p* = 0.001) independently predicted surgical difficulty level in multivariate logistic regression analysis. The nomogram model combining these predictors had an area under the ROC curve (AUC) of 0.801 (95% CI: 0.719–0.868) for the prediction of a high level of surgical difficulty. The DCA suggested that using the nomogram to predict surgical difficulty provided a clinical benefit.

**Conclusions:**

The nomogram model is feasible for predicting the difficulty level of rectal surgery, utilizing MRI-based pelvimetry parameters and clinical factors in mid to low RC cases.

## Introduction

Rectal cancer (RC) transabdominal resection is under the principle of total mesorectal excision (TME) (1). The standard procedure of radical excision reduces the positive circumferential margin and local recurrence rates, and the quality of surgery directly impacts local tumor control and patient prognosis (2). Even after treatment by a surgeon experienced in RC surgery, poor-quality rectal TME surgery is found in 15%–50% of cases, particularly in mid to low RC cases (3–5).

Open surgery allows for better exposure to the surgical field and provides tactile sensation to facilitate the stereo-visual assessment to precisely remove the lesion. The quality of open resection depends on the surgeon’s skills as well as on parameters associated with the patient, e.g., challenging anatomical conditions, including a narrow pelvis. A narrow pelvis is a pejorative factor leading to surgical challenges and increasing the odds of non-curative resection, largely because of the narrow space of the pelvic cavity limiting surgical access and visualization (6–9). Additional factors, e.g., male gender, obesity, and tumor characteristics, exacerbate surgical difficulty and thus decrease prognosis (10–12).

Preoperative MRI is increasingly utilized for predicting surgical difficulty, as MRI is widely applied in routine preoperative evaluation of RC (13–16). Several reports have indicated that bony pelvimetry measurements by MRI may predict the surgical difficulty related to TME (17–20). Conversely, several reports have found no association between MRI pelvimetry and surgical difficulty (21–23). It is challenging to compare the data among these reports, as they included distinct outcomes, pelvic measurements, surgical techniques, and imaging approaches, and it may not be accurate and convenient to adopt MRI pelvimetry in clinical practice (17). Therefore, the sole application of pelvimetry for predicting operative difficulty is an oversimplification.

Hence, developing a presurgical, non-invasive, and quantitative accurate strategy is required. A nomogram represents a graphical illustration of predictive statistical models for individual cases, with multiple advantages over the traditional logistic regression (24). To the best of our knowledge, no study has validated the short-term perioperative outcomes of open surgery for mid to low rectal cancer using an MRI pelvimetry nomogram. Thus, the present work aimed to assess the associations of MRI-based pelvimetry with surgical difficulty criteria in middle and low RC patients following transabdominal resection. Moreover, we developed an accurate predictive nomogram incorporating pelvic morphological and patient-related characteristics for predicting surgical difficulty level.

## Materials and methods

### Patients

All rectal cancer cases administered transabdominal resection in Changhai Hospital between June 2020 and August 2021 were enrolled in this trial. The Colorectal Surgery Department of Changhai Hospital is a tertiary referral center, which focuses on the treatment of colorectal cancer. All demographics, inpatient records, outpatient records, operational records, postoperative pathological reports, and medical images were carefully retrieved from our database. This study had approval from the Committee on Ethics of Biomedicine, Changhai Hospital (#CHEC2022-006).

### Eligibility criteria

The inclusion criteria were as follows: 1) single lesion within 10 cm from the anal verge on baseline MRI images, 2) pathologically confirmed rectal adenocarcinoma, 3) preoperative rectal MRI scan within 14 days prior to surgical resection in our hospital, 4) radical transabdominal resection of RC in our hospital, and 5) complete clinicopathological data in our database.

**Figure 1:**
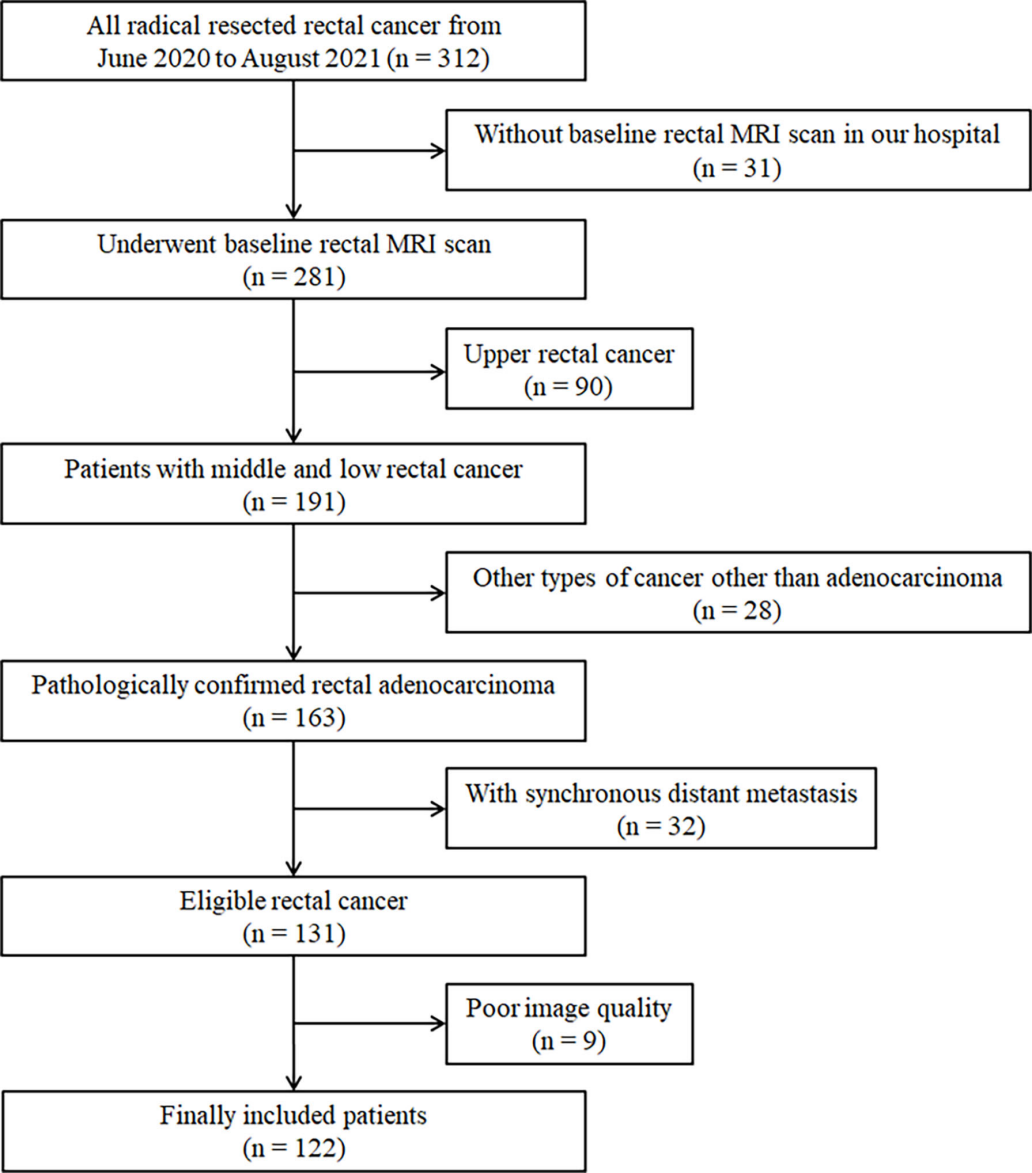
Patient selection flow diagram.

The exclusion criteria were as follows: 1) synchronous distant metastasis, (2) other types of cancer aside from adenocarcinoma, and (3) preoperative rectal MRI images too obscure to measure pelvimetry. The flowchart of the patient selection is shown in **Figure 1**.

### Operation and postoperative pathological assessment

All middle and low RC operations were performed by the same experienced surgical team (with a chief surgeon performing >300 RC operations annually), following the TME operation protocol, including abdominoperineal resection, low anterior resection, ultralow anterior resection, and intersphincteric resection. The decision on whether to perform an ileostomy was made at the chief surgeon’s discretion, according to cancer status and intraoperative situation. The stoma was reversed approximately 3–6 months after surgery.

Baseline patient characteristics such as age, gender, BMI, carcinoembryonic antigen (CEA, <5 ng/ml as negative), and carbohydrate antigen 19-9 (CA19-9, <37 U/ml as negative) were obtained from medical records. Pathologists checked the mesorectum dimension if intact. Following the National Comprehensive Cancer Network (NCCN) and American Joint Committee on Cancer (AJCC) TNM system (8th edition) (25), the resected specimens were examined by hematoxylin–eosin (H&E) staining. The ultimate reports comprised pathological TN staging, differentiation, and circumferential resection margin (CRM).

### Neoadjuvant chemoradiotherapy

In mid and low RC, neoadjuvant chemoradiotherapy (nCRT) is recommended for stage II/III tumors (1); however, some individuals decline this treatment. nCRT was applied at a total dose of 50 Gy in 25 fractions over 5 weeks. Concurrent chemotherapy with capecitabine alone (800 mg/m^2^ twice a day on 5 of 7 days in each radiotherapy week) or capecitabine with oxaliplatin (85 mg/m^2^ on day 1 of each week) was administered to the patients. Stage II/III rectal cancer cases not administered nCRT were recommended postsurgical CRT.

### Definition of operative difficulty

To examine the operative difficulty in RC patients following transabdominal resection, four criteria were selected for assessment: operative time, intraoperative blood loss, postoperative hospital stay, and postoperative complications. Each criterion was classified into two groups divided by the operative difficulty, representing a particular condition as follows: operative time, score = 0 (<120 min) and score = 1 (≥120 min); blood loss, score = 0 (<200 ml) and score = 1 (≥200 ml); postoperative hospital stay, score = 0 (<7 days) and score = 1 (≥7 days); and postoperative complications (graded according to the Clavien–Dindo classification), score = 0 (no) and score = 1 (yes).

Total score was cumulative as per surgical difficulty grade ranging from 0 to 4. A grade below 2 was considered to reflect low odds of operative difficulty (low level), whereas a grade ≥2 reflected elevated odds of surgical difficulty (high level).

### Radiological assessment

In this study, routine rectal MRI was carried out on a 1.5- or 3.0-T MRI system using an abdominal phase array coil, acquiring high-resolution T2-weighted images (T2WI) in the oblique axial (perpendicular to the main direction of the rectum with a lesion), coronal, and sagittal planes (14–16). All patients fasted for 4 h, and rectal cleaning was performed with 20 ml of glycerin enema before the MRI scan. In some patients, a warm ultrasound (US) gel was utilized for visualizing the intraluminal portion of the tumor, although carefully to prevent overdistension according to tumor location, i.e., 60–80 ml for a lesion in the mid–low rectum (16, 17, 26, 27). The detailed parameters of the main T2WI findings are shown in **Supplemental Table 1**.

Preoperative rectal MRI findings were reviewed retrospectively in our picture archiving and communication system (PACS). Mid and low RC was defined as a rectal lesion with the lower margin within 10 cm from the anal verge on sagittal T2WI images (1, 14–16).

**Figure 2:**
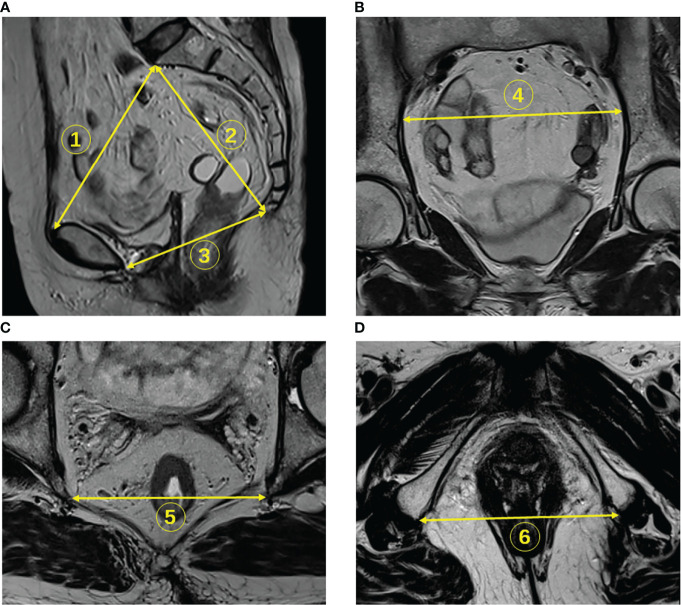
Identification of pelvimetry measurements on MR images. **(A)** The pelvic inlet (line 1), pelvic depth (line 2), and pelvic outlet (line 3) were measured on sagittal T2WI. **(B)** The transverse diameter was measured on coronal images (line 4). **(C)** The interspinous distance was measured on axial T2WI (line 5). **(D)** Intertuberous distance was measured on axial sections (line 6).

Pelvic inlet was defined with an anteroposterior diameter from the superior aspect of the pubic symphysis to the promontory, measured on the middle sagittal plane (**Figure 2A**, line 1). Pelvic depth was the distance from the promontory to the tailbone tip, assessed on the middle sagittal plane (**Figure 2A**, line 2). Pelvic outlet corresponded to the anteroposterior diameter from the inferior aspect of the pubic symphysis to the tailbone tip, assessed on the middle sagittal plane (**Figure 2A**, line 3). Transverse diameter was defined as the distance between the outermost points of the iliopectineal lines, assessed on the coronal plane (**Figure 2B**, line 4). Interspinous distance corresponded to the transverse distance between the tips of ischial spines, assessed on the axial plane (**Figure 2C**, line 5). Intertuberous distance corresponded to the transverse distance between the lowest points of ischial tuberosities, assessed on the axial plane (**Figure 2D**, line 6) (12, 18, 28–30).

All MRI-based pelvimetry measurements were carried out by two blinded radiologists in an independent manner (YY and ML with 10 and 8 years of experience in medical imaging, respectively). The values obtained by these radiologists were compared, and the mean value for each case was used in the final analysis.

### Statistical analysis

The normality of continuous data was determined by the Kolmogorov–Smirnov test. Continuous variables were presented as the mean ± standard deviation (SD), and categorical variables were presented as percentage. Categorical variables were compared using the chi-square test or Fisher’s exact test. Interobserver reproducibility for pelvimetry measurements was examined by determining intraclass correlation coefficients (ICCs), coefficients of variability (CVs), and Bland–Altman plots. ICC values >0.75, 0.4–0.75, and <0.4 were considered to indicate excellent, good, and poor agreement, respectively. The correlations of mean pelvimetry parameters with surgical difficulty grade were determined by the Spearman correlation test. Differences in pelvimetry indexes among difficulty groups were evaluated by the Kruskal–Wallis one-way ANOVA. Logistic regression analysis was performed to assess associations of various indexes with surgical difficulty level. Multivariable analysis was performed by stepwise logistic regression to determine the most influential predictive factors of surgical difficulty, which were used to develop a prediction nomogram model. The Hosmer–Lemeshow test was carried out to evaluate the model’s goodness of fit. Predictive performance was assessed by the receiver operating characteristic (ROC) curve analysis. Decision curve analysis (DCA) was performed for identifying the clinical usefulness of the predictive model by quantitating the standardized net benefits. The data were analyzed with SPSS 20.0 (SPSS, USA) and R v3.6.2. *p <*0.05 indicated statistical significance.

## Results

### Clinicopathological features

A total of 122 individuals operated for RC located in the middle and lower rectums were enrolled. The trial included 83 men and 39 women, aged 58.5 ± 10.8 years. BMI values at surgery were 24.0 ± 8.4 kg/m^2^. No patient had a positive resection margin after pathological analysis. Detailed clinicopathological features are presented in **Table 1**.

**Table 1:** Clinical and pathological characteristics in patients with rectal cancer.

Variable	No. of patients (*n* = 122)
Age (years)	58.5 ± 10.8 (27–83)
Sex (M/F)	83/39
BMI (kg/m^2^)	24.0 ± 8.4 (16.9–33.9)
CEA level	
≤5 ng/ml	78 (63.9)
>5 ng/ml	44 (36.1)
CA19-9 level	
≤37 U/ml	108 (88.5)
>37 U/ml	14 (11.5)
Operation history	
Yes	33 (27.0)
No	89 (73.0)
Neoadjuvant chemoradiotherapy	
Yes	17 (13.9)
No	105 (86.1)
Pathological T staging	
pT0 (pCR)	1 (0.8)
pT1	9 (7.4)
pT2	36 (29.5)
pT3	71 (58.2)
pT4	5 (4.1)
Pathological N staging	
pN0	60 (49.2)
pN1	33 (27.0)
pN2	29 (23.8)
Tumor location	
Middle	93 (76.2)
Low	29 (23.8)
Differentiation	
Well–moderate	105 (86.1)
Poor	17 (13.9)
Perineural invasion	
Positive	36 (29.5)
Negative	86 (70.5)
Tumor budding	
Bd 1	100 (82.0)
Bd 2	14 (11.5)
Bd 3	8 (6.5)
Lymphovascular invasion	
Positive	30 (24.6)
Negative	92 (75.4)
Tumor deposit	
Positive	37 (30.3)
Negative	85 (69.7)
Tumor size (mm)	25.2 ± 3.8 (18.3–38.5)

BMI, body mass index; CEA, carcinoembryonic antigen; CA19-9, carbohydrate antigen 19-9; pCR, pathologic complete response; Bd, budding.

### Surgical difficulty criteria and grade

The surgical data are listed in **Table 2**. The median operative time was 120 min, and 22 cases had a score of 1. The median blood loss was 200 ml, and 30 cases had a score of 1. The median postsurgical hospital stay was 7 days, and 66 cases had a score of 1. A total of 10 out of 122 patients with postoperative complications had a score of 1. Our results only included grade I and II surgical complications, including wound infections, urinary tract infections, transient elevation of serum creatinine, pulmonary infection, intra-abdominal abscess, and bowel obstruction. Surgical difficulty grades ranged between 0 and 4 points; in total, 36/122 (29.5%) and 86/122 (70.5%) cases were classified into the high-level and low-level groups, respectively.

**Table 2:** Surgical data.

	No. of patients (*n* = 122)
Surgical procedure	
Low anterior resection	114 (93.4)
Abdominoperineal resection	2 (1.6)
Ultralow anterior resection	3 (2.5)
Intersphincteric resection	3 (2.5)
Operative time (min)^a^	120 (IQR, 45)
Blood loss (ml)^a^	200 (IQR, 62.5)
Postoperative hospital stay (day)^a^	7 (IQR, 2)
Terminal ileostomy	
Yes	108 (88.5)
No	14 (11.5)
Postoperative complication	
No	112 (91.8)
Yes	
Grade I (wound infection and transient elevation of serum creatinine)	2 (1.6)
Grade II (urinary tract infection, pulmonary infection, intra-abdominal abscess, and bowel obstruction)	8 (6.6)

a Median (interquartile range).

### Consistency of MRI-based pelvimetry parameters between two radiologists

All ICCs for the six MRI-based pelvimetry parameters between the two radiologists indicated good consistency (range of 0.925 to 0.979). The CVs for the six MRI-based pelvimetry parameters between the two radiologists were all below 3%. Bland–Altman analysis of six MRI-based pelvimetry parameters by the two radiologists is shown in **Table 3** and **Supplemental Figure 1**.

**Table 3:** The consistency of MRI-based pelvimetry between two radiologists.

MRI-based pelvimetry	Mean ± SD (range)	ICC (95% CI)	CV (%)	Bias (LoA)
Pelvic inlet	82.3 ± 9.8 (61.2–116.2)	0.973 (0.961 to 0.981)	2.861	−0.787 (−7.158 to 5.585)
Pelvic depth	125.8 ± 14.1 (94.5–158.8)	0.974 (0.962 to 0.982)	2.741	−1.698 (−10.699 to 7.302)
Pelvic outlet	118.1 ± 11.6 (73.1–148.7)	0.963 (0.947 to 0.974)	2.751	−1.281 (−9.968 to 7.406)
Transverse diameter	128.5 ± 8.1 (108.7–145.7)	0.925 (0.893 to 0.948)	2.533	−1.364 (−10.015 to 7.288)
Interspinous distance	98.9 ± 9.9 (78.4–126.0)	0.968 (0.953 to 0.977)	2.743	−1.442 (−8.437 to 5.553)
Intertuberous distance	114.6 ± 13.0 (81.9–144.1)	0.979 (0.970 to 0.985)	2.489	−1.524 (−8.878 to 5.830)

ICC, intraclass correlation coefficient, between two observers; CI, confidence interval; CV, coefficient of variation; LoA, limit of agreement.

### Associations of clinicopathological factors with surgical difficulty criteria

The comparisons of categorical clinicopathological parameters in RC patients with the four surgical difficulty criteria, respectively, are shown in **Supplemental Table 2** and **Supplemental Figure 2**. Compared with the male gender, the female gender had associations with shorter operative time and shorter postsurgical hospital stay. Moreover, BMI had associations with operative time and postoperative complications, perineural invasion had an association with longer operative time, an association between larger tumor size and prolonged postsurgical hospital stay was found, intertuberous distance had an association with operative time, and there was an association of transverse diameter with postoperative hospital stay. All the above associations were statistically significant (all *p* < 0.05).

**Figure 3:**
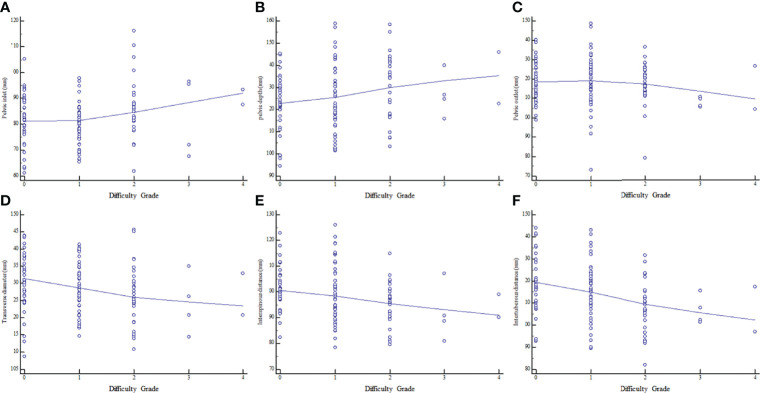
Relationships between surgical difficulty grade and MRI-based pelvimetry indexes. **(A)** Pelvic inlet. **(B)** Pelvic depth. **(C)** Pelvic outlet. **(D)** Transverse diameter. **(E)** Interspinous distance. **(F)** Intertuberous distance.

The associations of MRI-based pelvimetry parameters with difficulty grade are shown in **Supplemental Table 3**. Correlation analysis showed that pelvic inlet and pelvic depth were positively correlated with surgical difficulty grade; meanwhile, pelvic inlet, transverse diameter, interspinous distance, and intertuberous distance were negatively correlated with surgical difficulty grade (**Figure 3**).

### Univariable and multivariable regression analyses of associations between all factors and surgical difficulty level

In this study, sex, BMI, operation history, pelvic inlet, pelvic depth, transverse diameter, interspinous distance, and intertuberous distance were correlated with operative difficulty level in the univariate analysis (**Table 4**). Finally, BMI, pelvic inlet, and intertuberous distance independently predicted surgical difficulty level in the multivariable analysis (all *p* < 0.05) (**Table 5**). There was no factor correlated with operative difficulty level in the female subgroup (39 women, *p* > 0.05, **Supplemental Table 4**).

**Table 4:** Univariable logistic regression analyses of associations between factors and surgical difficulty level.

	Low level (*n* = 86)	High level (*n* = 36)	OR (95% CI)	*P*-value
Age (years)	59.3 ± 10.7	56.6 ± 11.1	0.977 (0.943, 1.013)	0.215
Sex				**0.001**
M	50	33	1 (reference)	
F	36	3	0.126 (0.036, 0.444)	
BMI (kg/m^2^)	23.4 ± 2.7	25.5 ± 3.0	1.331 (1.130, 1.569)	**0.001**
CEA level				0.995
≤5 ng/ml	55	23	1 (reference)	
>5 ng/ml	31	13	1.003 (0.446, 2.254)	
CA19-9 level				0.935
≤37 U/ml	76	32	1 (reference)	
>37 U/ml	10	4	0.950 (0.277, 3.253)	
Operation history				**0.040**
No	58	31	1 (reference)	
Yes	28	5	0.334 (0.117, 0.952)	
Neoadjuvant chemoradiotherapy				0.260
No	76	29	1 (reference)	
Yes	10	7	1.834 (0.638, 5.276)	
Tumor location				0.837
Middle	66	27	1 (reference)	
Low	20	9	1.100 (0.445, 2.720)	
Pathological T stage				0.294
≤T2	35	11	1 (reference)	
≥T3	51	25	1.560 (0.680, 3.575)	
Pathological N stage				0.178
Negative	45	14	1 (reference)	
Positive	41	22	1.725 (0.781, 3.810)	
Differentiation				0.257
Well–moderate	72	33	1 (reference)	
Poor	14	3	0.468 (0.126, 1.738)	
Perineural invasion				0.870
Negative	61	25	1 (reference)	
Positive	25	11	1.074 (0.460, 2.507)	
Tumor budding				
Bd 1	72	28	1 (reference)	
Bd 2	8	6	1.929 (0.614, 6.060)	0.261
Bd 3	6	2	0.857 (0.163, 4.503)	0.855
Lymphovascular invasion				0.059
Negative	69	23	1 (reference)	
Positive	17	13	2.294 (0.968, 5.436)	
Tumor deposit				0.641
Negative	61	24	1 (reference)	
Positive	25	12	1.220 (0.529, 2.811)	
Tumor size (mm)	25.0 ± 4.1	25.5 ± 3.0	1.029 (0.930, 1.137)	0.582
Pelvic inlet	80.6 ± 8.7	86.5 ± 11.2	1.066 (1.020, 1.114)	**0.004**
Pelvic depth	123.7 ± 13.8	131.0 ± 13.8	1.039 (1.006, 1.071)	**0.011**
Pelvic outlet	118.9 ± 12.0	116.2 ± 10.3	0.980 (0.947, 1.014)	0.238
Transverse diameter	129.5 ± 7.8	126.1 ± 8.3	0.947 (0.900, 0.996)	**0.034**
Interspinous distance	100.5 ± 10.1	95.0 ± 8.3	0.938 (0.896, 0.982)	**0.006**
Intertuberous distance	117.3 ± 12.8	108.3 ± 11.1	0.941 (0.908, 0.975)	**0.001**

OR, odds ratio.

The bold values P-value <0.05.

**Table 5:** Multivariable logistic regression analyses of associations between predictive factors and surgical difficulty level.

	OR (95% CI)	*P*-value
Sex (M/F)	0.238 (0.049, 1.151)	0.074
BMI (kg/m^2^)	1.255 (1.042, 1.510)	**0.017**
Operation history	0.621 (0.175, 2.205)	0.462
Pelvic inlet	1.070 (1.014, 1.130)	**0.014**
Pelvic depth	1.031 (0.991, 1.073)	0.133
Transverse diameter	0.969 (0.908, 1.035)	0.346
Interspinous distance	1.026 (0.950, 1.109)	0.515
Intertuberous distance	0.943 (0.891, 0.997)	**0.041**

OR, odds ratio.

The bold values P-value <0.05.

### The Nomogram Model

**Figure 4:**
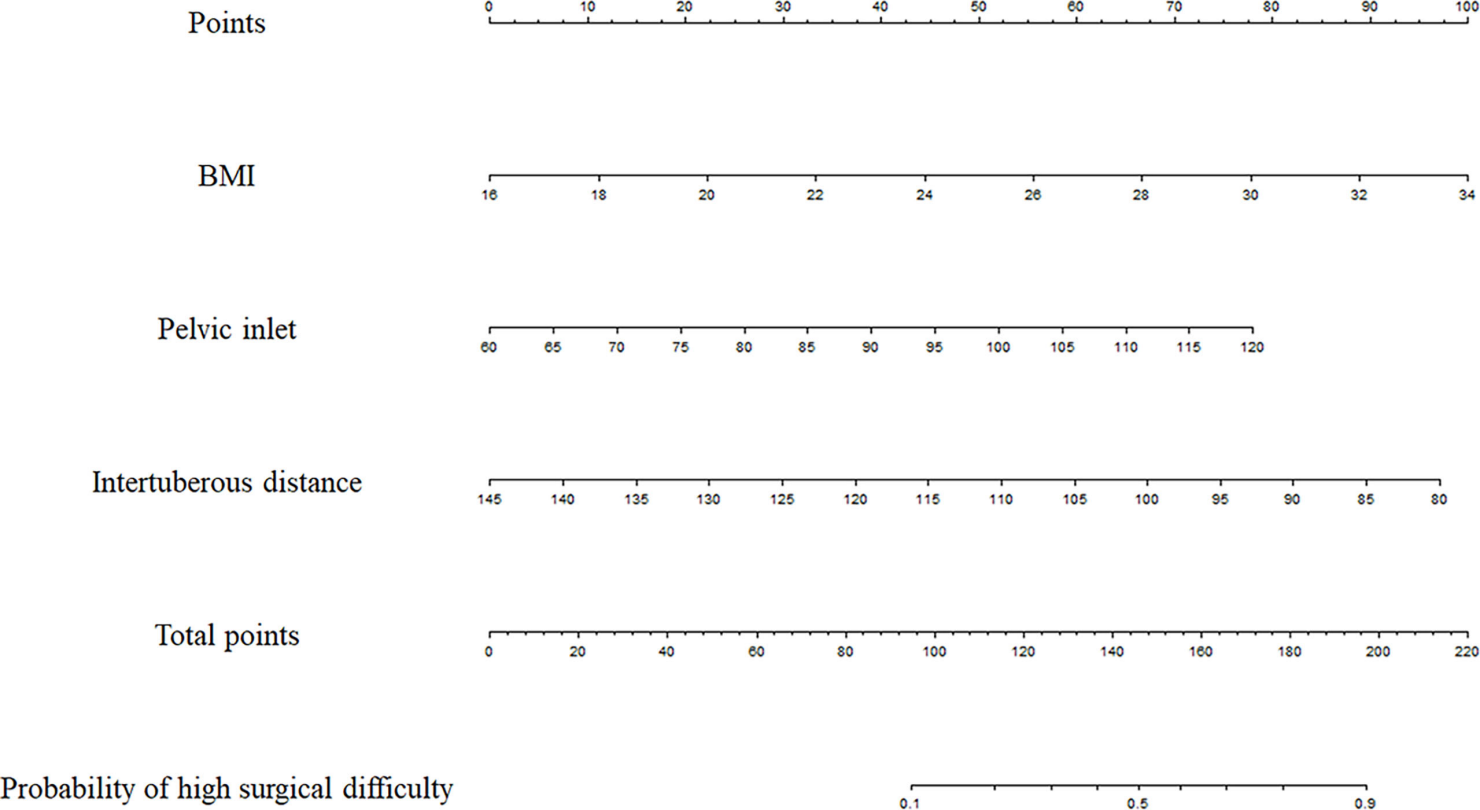
Prediction nomogram. In the nomogram, first, a vertical line was drawn according to the value of the most influential factors to determine the corresponding numbers of points. The total points were the sum of the above points. Then, a vertical line was drawn according to the value of total points to determine the probability of high surgical difficulty.

The three factors with significant associations with surgical difficulty in the multivariate analysis were utilized to develop the following model: probability of high surgical difficulty = −4.128 + 0.238 × BMI + 0.056 × pelvic inlet − 0.064 × intertuberous distance. The cutoff point was 0.344. The Hosmer–Lemeshow test showed a favorable model calibration (*p* = 0.974). A nomogram model was constructed combining the determined risk factors (BMI, OR = 1.269, *p* = 0.006; pelvic inlet, OR = 1.057, *p* = 0.024; and intertuberous distance, OR = 0.938, *p* = 0.001) (**Figure 4**).

**Figure 5:**
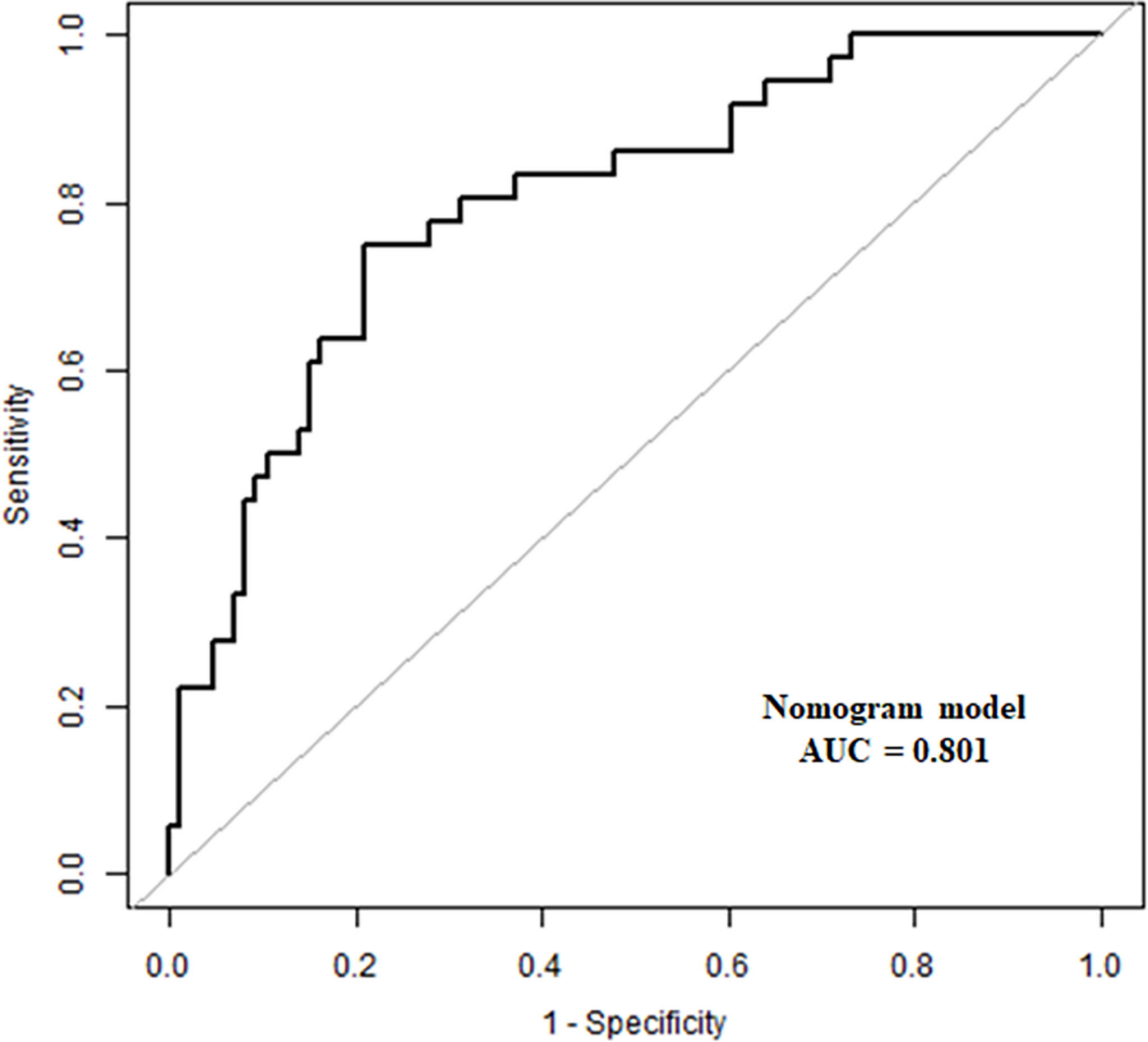
Receiver operating characteristic (ROC) curve. The AUC was 0.801 (95% CI, 0.719–0.868).

**Figure 6:**
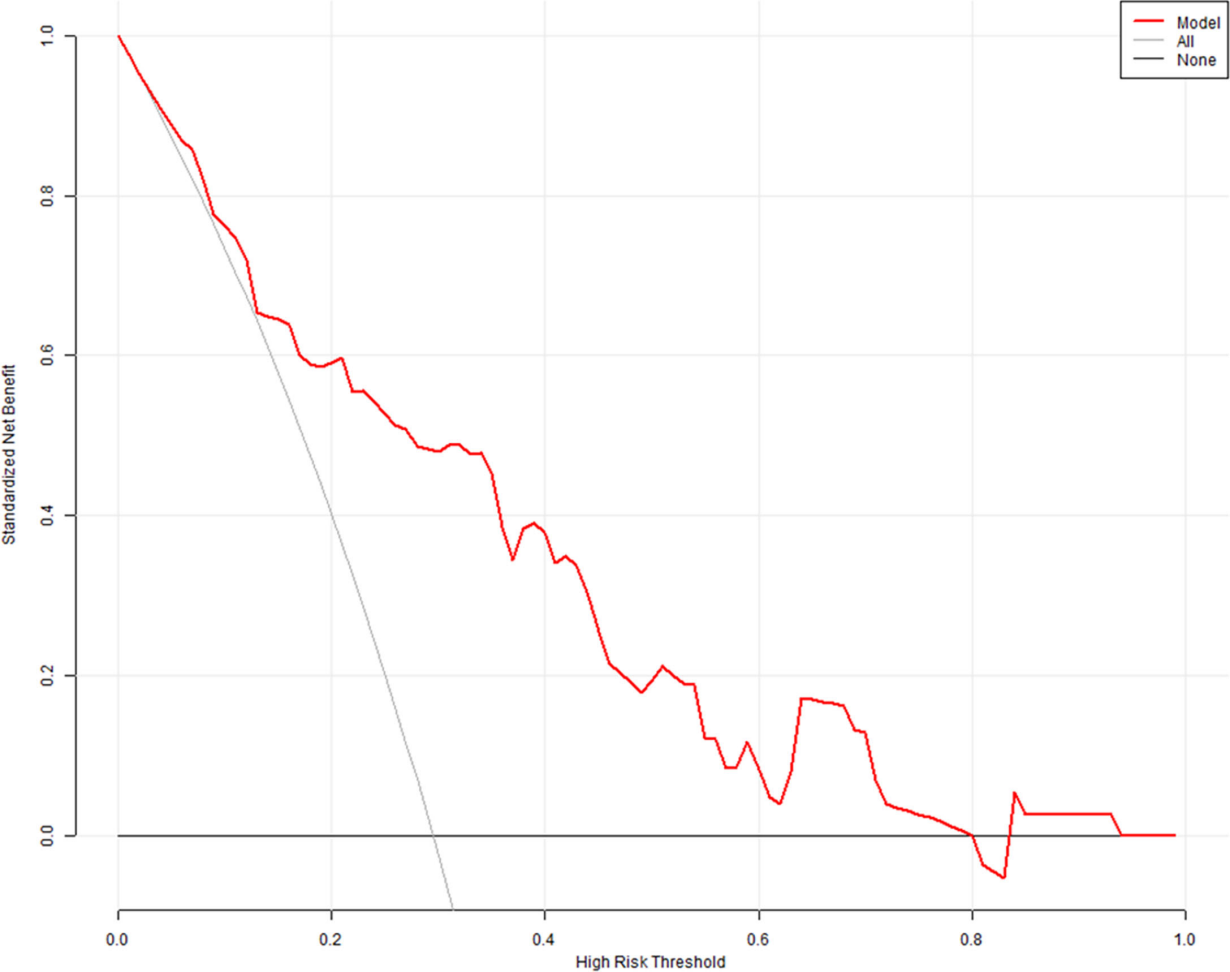
Decision curve analysis (DCA). The *x*- and *y*-axes represent the threshold probability and standardized net benefit, respectively. Red, gray, and black lines represent data acquired with the prediction nomogram model, the assumption that all patients had a high risk of difficult surgery, and the assumption that no patients had a high risk of difficult surgery, respectively.

The predictive model had an area under the ROC curve (AUC) of 0.801 (95% CI: 0.719–0.868) for the prediction of high surgical difficulty level (**Figure 5**); the sensitivity was 0.750 and the specificity was 0.791. The DCA of the nomogram is depicted in **Figure 6**. The DCA showed that at a threshold probability below 80%, utilizing the developed nomogram for predicting the odds of high surgical difficulty level conferred a positive net benefit versus the all-or-none scheme.

## Discussion

In the current study, we demonstrated an association between MRI-based pelvimetry and surgical difficulty in patients administered transabdominal resection of mid and low RC. Multivariate logistic regression analysis demonstrated that BMI, pelvic inlet, and intertuberous distance independently predicted surgical difficulty. Then, a nomogram was constructed with the selected predictors, which might help identify patients at risk of difficult surgery.

TME represents a standard procedure in radical RC surgery, which decreases the positive radial margin as well as local recurrence. Surgical difficulty was associated with surgical time, intraoperative blood loss, postoperative hospital stay, and complications in this study. Although pelvimetry has demonstrated association with surgical difficulty in RC patients, quantitative analyses examining the associations of pelvic anatomy with operative data are inconclusive (28–31). Conversely, many studies found no associations between pelvic measurements and surgical difficulty criteria (21–23). However, some reliable patient- and tumor-associated indexes might help determine surgical difficulty (17–20). Our results showed that BMI index, pelvic inlet, and intertuberous distance had associations with surgical difficulty, partially consistent with previously published findings.

Traditionally, the BMI represents the most common index utilized to describe overall obesity because it is easily obtained. A high BMI is a predictor of high-grade surgical difficulty (32), particularly in obese male subjects (33). In the clinic, we found that a high BMI is frequently associated with elevated odds of postsurgical complications in RC cases (34). However, BMI might not accurately reflect changes in visceral fat or overall obesity. Perirectal fat, as part of the visceral fat surrounding the rectum within the mesorectal fascia (MRF), is speculated to actively affect RC development (35, 36). However, mesorectal area or volume measurements were not calculated in this study because it is hard to avoid bias associated with rectal filling. Whether the rectal lumen should be distended with fluid or gel before MRI remains unclear (13). Studies (13–16) have reported filling the rectum for better visualization of lesions and evaluation of tumor penetration on MRI. In contrast, other studies (37–39) advised against distending the rectum due to possible undesirable effects on the distance between the rectal lesion and MRF. Therefore, the mesorectal area or volume was not included in this study due to instability.

The multivariable analysis also indicated that pelvic inlet and intertuberous distance had significant associations with surgical difficulty in RC patients as relevant variables. Pelvic inlet represents the anteroposterior diameter of the superior aperture of the pelvis, and intertuberous distance represents the transverse diameter of the inferior aperture of the pelvis. Our results indicated that shorter intertuberous distance could help predict high surgical difficulty similar to previously reported findings. Conversely, we identified a positive association between pelvic inlet and operative difficulty, which is inconsistent with most research findings (17–20). However, this finding is partially similar to a study by Shimada et al. (28). They found that the anteroposterior diameter/transverse diameter ratio of the pelvis was correlated with operative difficulty. This phenomenon could be attributed to the longer anteroposterior diameter of the pelvic inlet, and the reduced transverse diameter of the outlet might represent the anthropoid-type pelvic shape, which is vertically deeper and transversally narrower than other pelvis types (40). Thus, pelvic inlet incorporated with intertuberous distance might provide clinical measurements for predicting the pelvis with surgical difficulty.

Furthermore, a nomogram model was built in the present work to predict operative difficulty, incorporating independent predictors from the multivariable analysis. ROC analysis and DCA proposed that the practical nomogram model may have a great value as a predictive visualization tool in RC. The nomogram could be used to easily assess individuals undergoing surgery for operative difficulty level.

The limitations of this study should be mentioned. Firstly, it was a small-sample retrospective study performed in a single institution, and selection bias could not be avoided. In addition, we did not assess long-term oncologic outcomes, including recurrence, morbidity, and mortality rates. Therefore, prospective randomized studies with larger samples and longer follow-up are warranted. Secondly, this work did not include upper RC (URC) cases in the analysis, which shows a decreased risk of CRM involvement and incomplete TME (41, 42), one of the potential reasons why upper rectal lesions are presumably less affected by the shape of the pelvis (12). Whether these findings are applicable to URC requires further research. Thirdly, as our treating surgeon was experienced in RC surgery, specifically open resection, transanal or laparoscopic surgeries were not included since there were too few relevant cases for a meaningful analysis. The benefits and popularity of minimally invasive surgery are undeniable around the globe although the laparoscopic approach in the treatment of middle or low rectal cancer remains controversial. Studies have shown differences between open surgery and minimally invasive proctectomy in surgical difficulty criteria (3, 43–46). Therefore, the current findings should be verified in patients operated by other minimally invasive approaches. Fourthly, only 2D MRI-based pelvimetry was utilized to construct the nomogram, not including 3D features, which have the potential to be utilized in multiple aspects of pelvis shape. However, 3D reconstruction in pelvimetry requires expensive software, complex techniques, and a long time (28, 29, 31). Thus, 3D techniques are difficult and not convenient to adopt in the clinic. Further assessment of 3D pelvimetry should be conducted to define surgical difficulty based on pelvis features.

## Conclusions

In this study, we built a nomogram prediction model including both clinical variables and MRI-based pelvimetry data. This objective method would provide a visualization tool to effectively predict the probability of surgical difficulty in RC.

## Data availability statement

The raw data supporting the conclusions of this article will be made available by the authors, without undue reservation.

## Ethics statement

Written informed consent was obtained from the individual(s) for the publication of any potentially identifiable images or data included in this article.

## Author contributions

FS and XS: conceptualization. YY, DT, and ML: preparation of the original draft, data collection, and statistical analysis. FS, HL, and XS: funding acquisition. YY and DT: manuscript writing. FS and XS: supervision and manuscript review and editing. All authors contributed to the article and approved the submitted version.

## Funding

This study was funded by the 234 Subject Climbing Plan of Changhai Hospital (#2020YZL005) and the Changhai Hospital Discipline Construction Project (#2020YXK034). The funders developed the main idea and designed the study.

## Conflict of interest

The authors declare that the research was conducted in the absence of any commercial or financial relationships that could be construed as a potential conflict of interest.

## Publisher’s note

All claims expressed in this article are solely those of the authors and do not necessarily represent those of their affiliated organizations, or those of the publisher, the editors and the reviewers. Any product that may be evaluated in this article, or claim that may be made by its manufacturer, is not guaranteed or endorsed by the publisher.

## Supplementary material

Supplementary Figure 1 Bland-Altman graphs. The solid blue line indicates the mean difference in reads between the two radiologists. The dashed red lines indicate the limits of agreement (LoA). **(A)**. Pelvic inlet. **(B)**. Pelvic depth. **(C)**. Pelvic outlet. **(D)**. Transverse diameter. **(E)**. Interspinous distance. F. Intertuberous distance.

Supplementary Figure 2 Boxplots of pelvimetry based on surgical difficulty criteria. **(A)**. Operative time. **(B)**. Intraoperative blood loss. **(C)**. Postoperative hospital stay. **(D)**. Postoperative complication. Line 1. Pelvic inlet. Line 2. Pelvic depth. Line 3. Pelvic outlet. Line 4. Transverse diameter. Line 5. Interspinous distance. Line 6. Intertuberous distance. **p* < 0.05, Comparison between two groups.

## Abbreviations

RC, rectal cancer; TME, total mesorectal excision; ICC, intraclass correlation coefficient; T2WI, T2-weighted imaging; BMI, body mass index; nCRT, neoadjuvant chemoradiotherapy; ROC, receiver operating characteristic; OR, odds ratio; DCA, decision curve analysis.
