# Supplementary figures and images for: An MRI-based pelvimetry nomogram for predicting surgical difficulty of transabdominal resection in patients with middle and low rectal cancer
Source: Front Oncol. 2022 Jul 25;12:882300. doi: 10.3389/fonc.2022.882300 (PMC9357897; doi:10.3389/fonc.2022.882300)

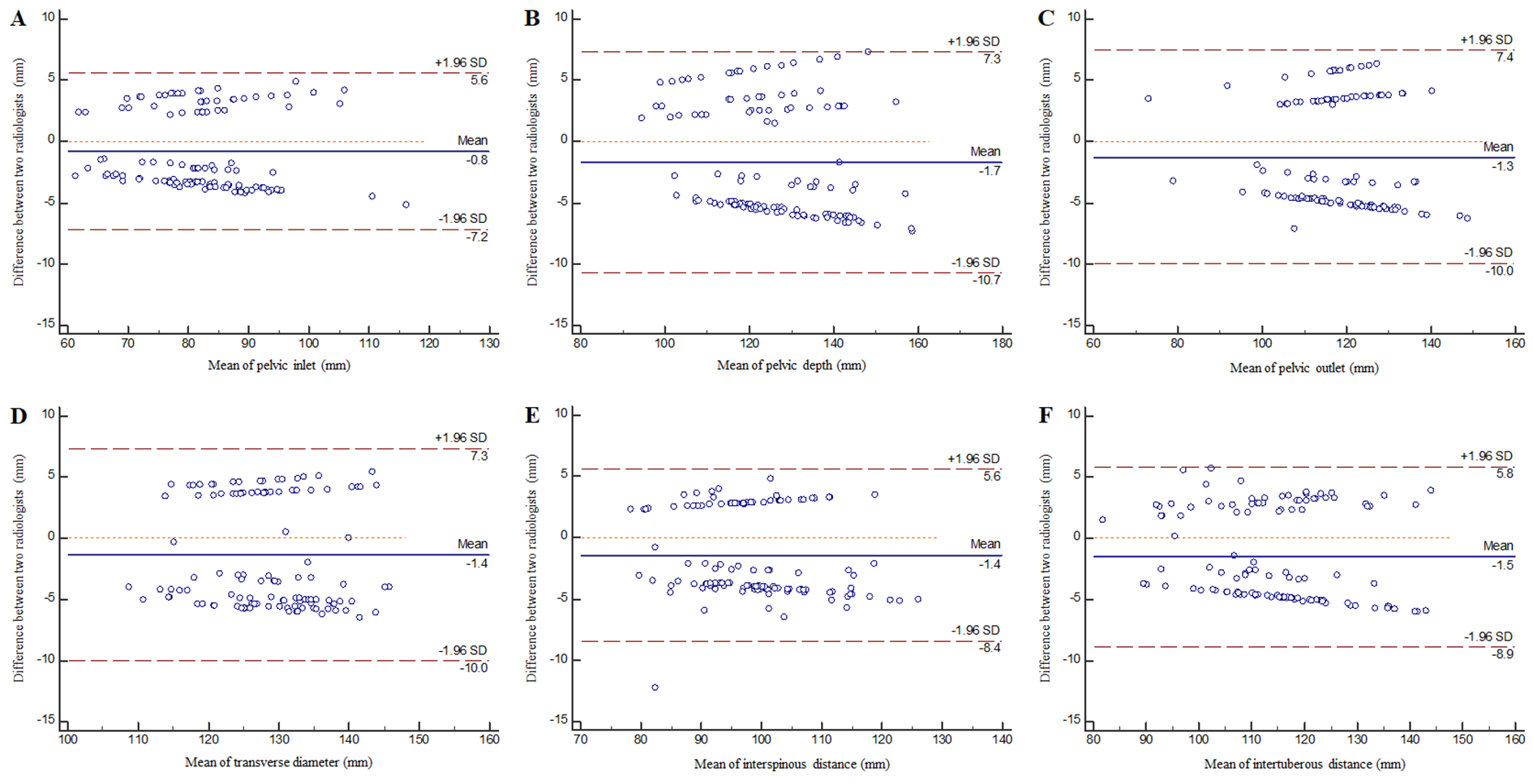

Supplement: Supplementary Figure 1 — Bland-Altman graphs. The solid blue line indicates the mean difference in reads between the two radiologists. The dashed red lines indicate the limits of agreement (LoA). (A). Pelvic inlet. (B). Pelvic depth. (C). Pelvic outlet. (D). Transverse diameter. (E). Interspinous distance. F. Intertuberous distance. [file Image_1.tif]

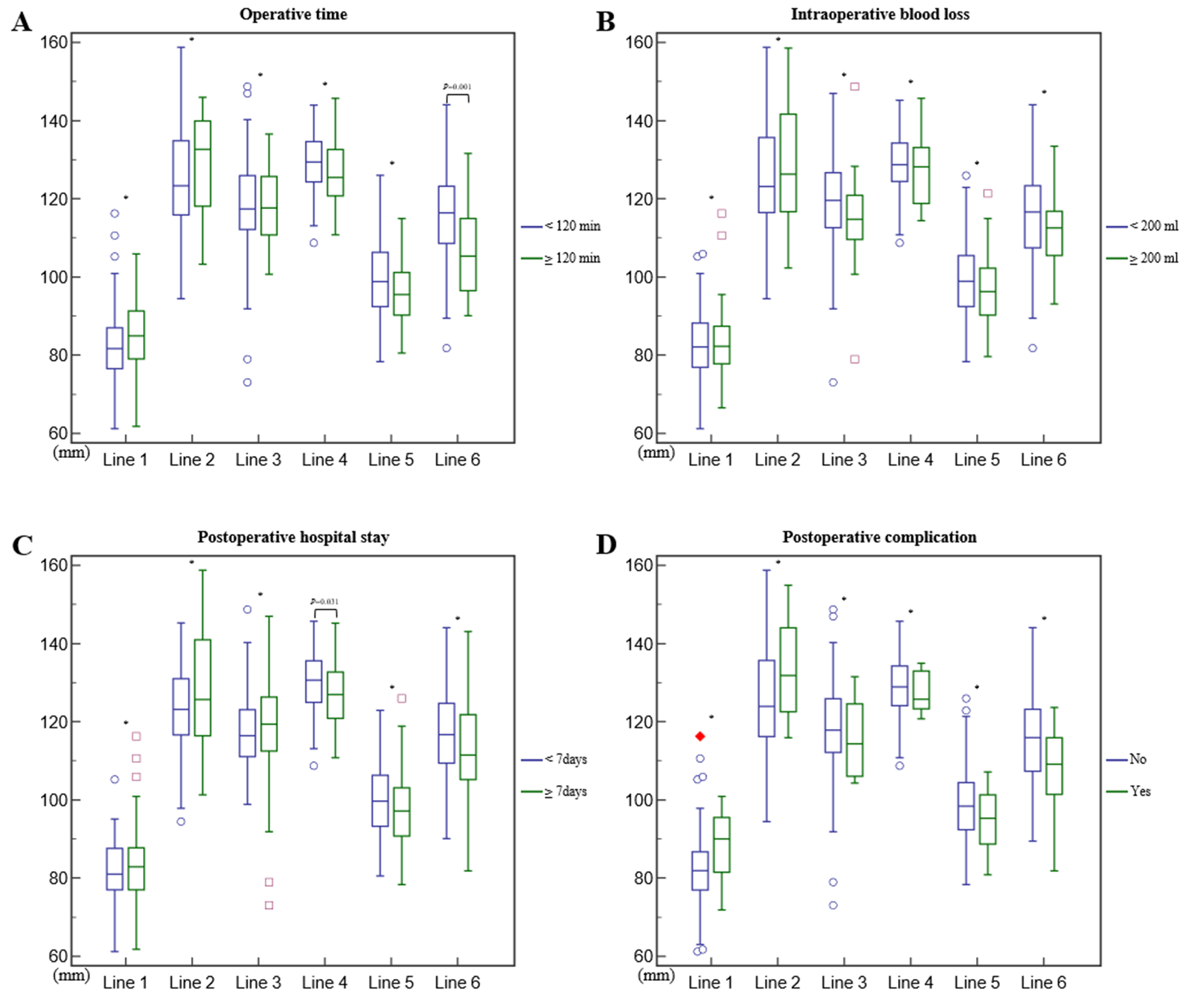

Supplement: Supplementary Figure 2 — Boxplots of pelvimetry based on surgical difficulty criteria. (A). Operative time. (B). Intraoperative blood loss. (C). Postoperative hospital stay. (D). Postoperative complication. Line 1. Pelvic inlet. Line 2. Pelvic depth. Line 3. Pelvic outlet. Line 4. Transverse diameter. Line 5. Interspinous distance. Line 6. Intertuberous distance. *p < 0.05, Comparison between two groups. [file Image_2.tif]
