# Supplementary material for: An MRI-based pelvimetry nomogram for predicting surgical difficulty of transabdominal resection in patients with middle and low rectal cancer
Source: Front Oncol. 2022 Jul 25;12:882300. doi: 10.3389/fonc.2022.882300 (PMC9357897; doi:10.3389/fonc.2022.882300)
Supplement: Supplementary file 3 [file Table_1.docx]

**Supplemental Table 1.** The main sequence protocol and parameters of rectal MRI

| **Scanner** | **GE**  **Discovery 750** | **GE**  **Signa HDX** | **SIEMENS**  **Skyra** | **SIEMENS**  **Avanto** |
| --- | --- | --- | --- | --- |
| **Magnetic field strength** | 3.0T | 3.0T | 3.0T | 1.5T |
| **Sagittal T2WI** |  |  |  |  |
| FOV (mm) | 280 | 230 | 230 | 230 |
| Thickness (mm) | 5 | 5 | 5 | 6 |
| Matrix | 320×320 | 320×320 | 320×320 | 256×256 |
| TR/TE (ms) | 4500/98 | 4275/106 | 5000/106 | 3800/91 |
| **Oblique axial T2WI** |  |  |  |  |
| FOV (mm) | 200 | 180 | 180 | 180 |
| Thickness (mm) | 4 | 3 | 3 | 3 |
| Matrix | 352×352 | 288×288 | 320×320 | 320×320 |
| TR/TE (ms) | 6538/116 | 4000/108 | 4000/108 | 4000/90 |
| **Coronal T2WI** |  |  |  |  |
| FOV (mm) | 230 | 230 | 230 | 230 |
| Thickness (mm) | 3 | 3 | 3 | 4.5 |
| Matrix | 320×320 | 320×320 | 384×384 | 384×384 |
| TR/TE (ms) | 5000/106 | 5000/106 | 4690/99 | 5800/99 |

FOV: field of view

TR/TE: repetition time/echo time.
