# Supplementary material for: An MRI-based pelvimetry nomogram for predicting surgical difficulty of transabdominal resection in patients with middle and low rectal cancer
Source: Front Oncol. 2022 Jul 25;12:882300. doi: 10.3389/fonc.2022.882300 (PMC9357897; doi:10.3389/fonc.2022.882300)
Supplement: Supplementary file 4 [file Table_2.docx]

**Supplemental Table 2. Univariable logistic regression analyses of associations between all factors and surgical difficulty criteria**

|  | **Operative time** | | **Intraoperative blood loss** | | **Postoperative hospital stay** | | | **Postoperative complication** | | | |  |
| --- | --- | --- | --- | --- | --- | --- | --- | --- | --- | --- | --- | --- |
|  | OR (95%CI) | *P* value | OR (95%CI) | *P* value | | OR (95%CI) | *P* value | OR (95%CI) | | *P* value | |  |
| Age (years) | 1.000 (0.958, 1.043) | 0.986 | 0.993 (0.956, 1.032) | 0.729 | | 0.976 (0.943, 1.010) | 0.157 | 0.994 (0.937, 1.056) | | 0.856 | |  |
| Sex |  | **0.021** |  | 0.475 | |  | **0.019** |  | | 0.154 | |  |
| M | 1 (reference) |  | 1 (reference) |  | | 1 (reference) |  | 1 (reference) | |  | |  |
| F | 0.170 (0.038, 0.770) |  | 0.716 (0.286, 1.791) |  | | 0.392 (0.179, 0.857) |  | 0.216 (0.026, 1.772) | |  | |  |
| BMI index (kg/m^2^) | 1.210 (1.024, 1.429) | **0.025** | 1.097 (0.951, 1.267) | 0.204 | | 1.063 (0.938, 1.205) | 0.337 | 1.345 (1.070, 1.692) | | **0.011** | |  |
| CEA level |  | 0.832 |  | 0.309 | |  | 0.235 |  | | 0.337 | |  |
| ≤5 ng/ml | 1 (reference) |  | 1 (reference) |  | | 1 (reference) |  | 1 (reference) | |  | |  |
| >5 ng/ml | 0.995 (0.949, 1.043) |  | 1.018 (0.984, 1.053) |  | | 1.030 (0.981, 1.081) |  | 0.920 (0.777, 1.090) | |  | |  |
| CA19-9 level |  | 0.726 |  | 0.927 | |  | 0.708 |  | | 0.556 | |  |
| ≤37 U/ml | 1 (reference) |  | 1 (reference) |  | | 1 (reference) |  | 1 (reference) | |  | |  |
| >37 U/ml | 1.001 (0.996, 1.006) |  | 1.000 (0.995, 1.006) |  | | 1.001 (0.996, 1.006) |  | 0.990 (0.958, 1.023) | |  | |  |
| Operation history |  | 0.615 |  | **0.023** | |  | 0.245 |  | | 0.827 | |  |
| No | 1 (reference) |  | 1 (reference) |  | | 1 (reference) |  | 1 (reference) | |  | |  |
| Yes | 0.756 (0.255, 2.246) |  | 0.230 (0.065, 0.817) |  | | 0.621 (0.278, 1.387) |  | 1.171 (0.284, 4.826) | |  | |  |
| Neoadjuvant therapy |  | 0.964 |  | 0.274 | |  | 0.149 |  | | 0.566 | |  |
| No | 1 (reference) |  | 1 (reference) |  | | 1 (reference) |  | 1 (reference) | |  | |  |
| Yes | 0.970 (0.253, 3.713) |  | 1.841 (0.616, 5.498) |  | | 2.267 (0.746, 6.887) |  | 1.617 (0.313, 8.352) | |  | |  |
| Tumor location |  | 0.498 |  | 0.358 | |  | 0.326 |  | | 0.219 | |  |
| Middle | 1 (reference) |  | 1 (reference) |  | | 1 (reference) |  | 1 (reference) | |  | |  |
| Low | 0.667 (0.206, 2.157) |  | 1.543 (0.612, 3.890) |  | | 1.534 (0.654, 3.601) |  | 2.320 (0.607, 8.870) | |  | |  |
| Pathological T stage |  | 0.117 |  | 0.318 | |  | 0.966 |  | | 0.407 | |  |
| ≤T2 | 1 (reference) |  | 1 (reference) |  | | 1 (reference) |  | | 1 (reference) | |  | |
| ≥T3 | 2.363 (0.807, 6.914) |  | 1.570 (0.648, 3.804) |  | | 0.984 (0.472, 2.052) |  | | 0.577 (0.158, 2.114) | |  | |
| Pathological N stage |  | 0.700 |  | 0.461 | |  | 0.210 | |  | | 0.073 | |
| Negative | 1 (reference) |  | 1 (reference) |  | | 1 (reference) |  | | 1 (reference) | |  | |
| Positive | 1.200 (0.475, 3.030) |  | 1.366 (0.596, 3.132) |  | | 1.582 (0.773, 3.240) |  | | 4.296 (0.873, 21.136) | |  | |
| Differentiation |  | 0.191 |  | 0.477 | |  | 0.531 | |  | | 0.566 | |
| Well-Moderate | 1 (reference) |  | 1 (reference) |  | | 1 (reference) |  | | 1 (reference) | |  | |
| Poor | 0.250 (0.031, 1.993) |  | 0.619 (0.165, 2.321) |  | | 0.720 (0.258, 2.012) |  | | 1.617 (0.313, 8.352) | |  | |
| Perineural invasion |  | **0.023** |  | 0.315 | |  | 0.834 | |  | | 0.972 | |
| Negative | 1 (reference) |  | 1 (reference) |  | | 1 (reference) |  | | 1 (reference) | |  | |
| Positive | 3.000 (1.160, 7.761) |  | 2.025 (0.511, 8.025) |  | | 1.087 (0.497, 2.376) |  | | 1.026 (0.250, 4.212) | |  | |
| Tumor budding |  |  |  |  | |  |  | |  | |  | |
| Bd 1 | 1 (reference) |  | 1 (reference) |  | | 1 (reference) |  | | 1 (reference) | |  | |
| Bd 2 | 1.953 (0.548, 6.965) | 0.302 | 0.885 (0.102, 7.659) | 0.911 | | 1.136 (0.367, 3.513) | 0.825 | | 0.885 (0.102, 7.659) | | 0.911 | |
| Bd 3 | 0.697 (0.080, 6.044) | 0.744 | 0.000 (0.000, Inf) | 0.994 | | 0.852 (0.202, 3.598) | 0.827 | | 1.643 (0.179, 15.072) | | 0.661 | |
| Lymphovascular invasion |  | 0.162 |  | 0.864 | |  | 0.456 | |  | | 0.679 | |
| Negative | 1 (reference) |  | 1 (reference) |  | | 1 (reference) |  | | 1 (reference) | |  | |
| Positive | 2.026 (0.753, 5.448) |  | 0.867 (0.170, 4.420) |  | | 1.375 (0.595, 3.176) |  | | 1.349 (0.326, 5.583) | |  | |
| Tumor deposit |  | 0.867 |  | 0.839 | |  | 0.434 | |  | | 0.981 | |
| Negative | 1 (reference) |  | 1 (reference) |  | | 1 (reference) |  | | 1 (reference) | |  | |
| Positive | 1.089 (0.403, 2.942) |  | 1.162 (0.274, 4.918) |  | | 1.367 (0.625, 2.988) |  | | 0.983 (0.240, 4.032) | |  | |
| Tumor size (mm) | 1.018 (0.904, 1.146) | 0.770 | 0.975 (0.871, 1.092) | 0.665 | | 1.138 (1.019, 1.271) | **0.022** | | 1.085 (0.937, 1.256) | | 0.276 | |
| Pelvic inlet | 1.029 (0.982, 1.078) | 0.225 | 1.015 (0.973, 1.058) | 0.490 | | 1.020 (0.983, 1.059) | 0.287 | | 1.060 (0.996, 1.129) | | 0.065 | |
| Pelvic depth | 1.019 (0.986, 1.053) | 0.269 | 1.013 (0.983, 1.043) | 0.397 | | 1.022 (0.996, 1.049) | 0.102 | | 1.040 (0.992, 1.091) | | 0.105 | |
| Pelvic outlet | 0.994 (0.956, 1.034) | 0.775 | 0.966 (0.931, 1.002) | 0.064 | | 1.004 (0.973, 1.035) | 0.809 | | 0.979 (0.929, 1.033) | | 0.439 | |
| Transverse diameter | 0.962 (0.908, 1.020) | 0.196 | 0.973 (0.924, 1.025) | 0.307 | | 0.951 (0.907, 0.996) | **0.034** | | 0.976 (0.901, 1.058) | | 0.558 | |
| Interspinous distance | 0.956 (0.908, 1.006) | 0.084 | 0.972 (0.931, 1.016) | 0.209 | | 0.971 (0.936, 1.008) | 0.119 | | 0.953 (0.887, 1.025) | | 0.195 | |
| Intertuberous distance | 0.937 (0.898, 0.977) | **0.002** | 0.979 (0.948, 1.012) | 0.205 | | 0.976 (0.949, 1.004) | 0.098 | | 0.951 (0.901, 1.004) | | 0.068 | |

*OR*: odds ratio
