# Supplementary material for: An MRI-based pelvimetry nomogram for predicting surgical difficulty of transabdominal resection in patients with middle and low rectal cancer
Source: Front Oncol. 2022 Jul 25;12:882300. doi: 10.3389/fonc.2022.882300 (PMC9357897; doi:10.3389/fonc.2022.882300)
Supplement: Supplementary file 5 [file Table_3.docx]

**Supplemental Table 3. Analyses of associations between MRI-based pelvimetry and surgical difficulty grade**

| **MRI-based pelvimetry^†^** | **Grade 0 (n=38)** | **Grade 1 (n=48)** | **Grade 2 (n=30)** | **Grade 3 (n=4)** | **Grade 4 (n=2)** | ***P* value *** | ***ρ*** | ***P* value **** |
| --- | --- | --- | --- | --- | --- | --- | --- | --- |
| Pelvic inlet | 82.100 (61.200 – 105.250) | 80.100 (65.450 – 97.850) | 85.425 (61.800 – 116.200) | 83.750 (67.600 – 96.500) | 90.525 (87.600 – 93.450) | **0.018** | 0.155 | **0.088** |
| Pelvic depth | 123.150 (94.450 – 145.250) | 120.450 (101.300 – 158.750) | 135.300 (103.250 – 158.550) | 125.675 (115.900 – 139.900) | 134.250 (122.550 – 145.950) | 0.114 | 0.188 | **0.038** |
| Pelvic outlet | 117.250 (98.850 – 140.250) | 120.675 (73.050 – 148.650) | 117.000 (79.000 – 136.550) | 107.825 (105.500 – 110.750) | 115.450 (104.300 – 126.600) | 0.139 | -0.070 | 0.443 |
| Transverse diameter | 130.900 (108.700 – 143.950) | 129.000 (114.700 – 141.300) | 125.150 (110.800 – 145.700) | 123.475 (114.400 – 134.950) | 126.850 (120.750 – 132.950) | 0.137 | -0.237 | **0.009** |
| Interspinous distance | 99.675 (82.400 – 122.900) | 99.225 (78.350 – 126.000) | 96.075 (79.650 – 114.950) | 89.800 (80.850 – 107.150) | 94.600 (90.100 – 99.100) | 0.098 | -0.231 | **0.010** |
| Intertuberous distance | 117.650 (92.800 – 144.050) | 116.725 (89.450 – 143.050) | 107.425 (81.850 – 131.600) | 105.200 (101.400 – 115.650) | 107.225 (97.000 – 117.450) | **0.007** | -0.319 | **0.0003** |

† Median (range)

* Kruskal-Wallis test

** Spearman's test
