# Supplementary material for: An MRI-based pelvimetry nomogram for predicting surgical difficulty of transabdominal resection in patients with middle and low rectal cancer
Source: Front Oncol. 2022 Jul 25;12:882300. doi: 10.3389/fonc.2022.882300 (PMC9357897; doi:10.3389/fonc.2022.882300)
Supplement: Supplementary file 6 [file Table_4.docx]

**Supplemental Table 4. Subgroup analyses of associations between risk factors and surgical difficulty level**

|  | **Surgical difficulty level** | | | |
| --- | --- | --- | --- | --- |
|  | **Male (n=83)** | | **Female (n=39)** | |
|  | OR (95%CI) | *P* value | OR (95%CI) | *P* value |
| BMI index (kg/m^2^) | 1.367 (1.123, 1.665) | **0.002** | 1.159 (0.752, 1.788) | 0.504 |
| Operation history |  | 0.353 |  | 0.585 |
| No | 1 (reference) |  | 1 (reference) |  |
| Yes | 0.552 (0.157, 1.934) |  | 0.500 (0.042, 6.017) |  |
| Pelvic inlet | 1.090 (1.032, 1.150) | **0.002** | 1.057 (0.913, 1.224) | 0.457 |
| Pelvic depth | 1.018 (0.983, 1.054) | 0.315 | 1.074 (0.978, 1.179) | 0.136 |
| Transverse diameter | 0.974 (0.920, 1.031) | 0.357 | 1.029 (0.854, 1.241) | 0.762 |
| Interspinous distance | 0.971 (0.917, 1.028) | 0.316 | 0.982 (0.855, 1.128) | 0.798 |
| Intertuberous distance | 0.968 (0.929, 1.009) | 0.128 | 0.914 (0.817, 1.024) | 0.120 |

*OR*: odds ratio
